# Lottery Spending: A Non-Parametric Analysis

**DOI:** 10.1371/journal.pone.0115730

**Published:** 2015-02-02

**Authors:** Skip Garibaldi, Kayla Frisoli, Li Ke, Melody Lim

**Affiliations:** 1 Institute for Pure and Applied Mathematics, UCLA, 460 Portola Plaza, Box 957121, Los Angeles, California 90095-7121, USA; 2 Department of Mathematics & Computer Science, Emory University, 400 Dowman Dr, Atlanta, Georgia 30322, USA; University of East Piedmont, ITALY

## Abstract

We analyze the spending of individuals in the United States on lottery tickets in an average month, as reported in surveys. We view these surveys as sampling from an unknown distribution, and we use non-parametric methods to compare properties of this distribution for various demographic groups, as well as claims that some properties of this distribution are constant across surveys. We find that the observed higher spending by Hispanic lottery players can be attributed to differences in education levels, and we dispute previous claims that the top 10% of lottery players consistently account for 50% of lottery sales.

## Introduction

Lotteries are a big business in the United States, with revenue of $78 billion in fiscal year 2012 [1]. And they are a source of concern for many, from educators who rely on the proceeds for part of their funding, to those concerned about encouraging problem gamblers, and for others who worry that the lottery amounts to an implicit tax that is unfair to various demographic groups. In this note, we look at connections between demography and lottery spending using statistical tools that have not previously been used for this purpose, we use these tools to study newer data sets than previous studies, and we consider new properties of the distribution of spending.

Specifically, we are interested in how much a person chosen at random spends on lottery tickets in a typical month. There are many reasons to be interested in the distribution of this number, as well as how it differs for different demographic groups:

- The players who spend the most on the lottery account for a large fraction of total sales, and therefore are of interest to the lottery operators who want to understand them for marketing purposes.
- For states considering whether to adopt a lottery, it might be helpful for predicting the amount of revenue they might expect to bring in.
- The lottery “levies an implicit tax on players” [2], and the weight of that particular tax depends on the distribution of lottery spending. To what extent is this implicit tax regressive?
- Society has an interest in controlling pathological gambling. If a demographic group has a substantially higher incidence of heavy spending on the lottery, then this is a signal for pathological gambling.
- If a lottery operator has a good model of spending by heavy spenders, then they can use it as a baseline to detect excessive numbers of frequent prize claimants, which would indicate that some frequent prize claimants are engaged in (typically illegal) ticket aggregation schemes. For example, a recent investigation in Florida revealed that 8 of the top 10 prize claimants were participating in such schemes, see [3] and [4].

### Background and context

Many authors have previously analyzed who spends how much on the lottery, in some cases to understand whether the “implicit lottery tax” is regressive and in others to understand connections with incidence of problem gambling. That is, these studies have focused on which demographic groups spend more than others. See for example the books [2, 5]; the papers [6–13]; or the surveys [14, 15]. These papers have used various model-fitting techniques, such as tobit, Heckman-corrected tobit, truncated tobit, probit, or logit regression, and then looked at effect sizes.

**Fig 1:**
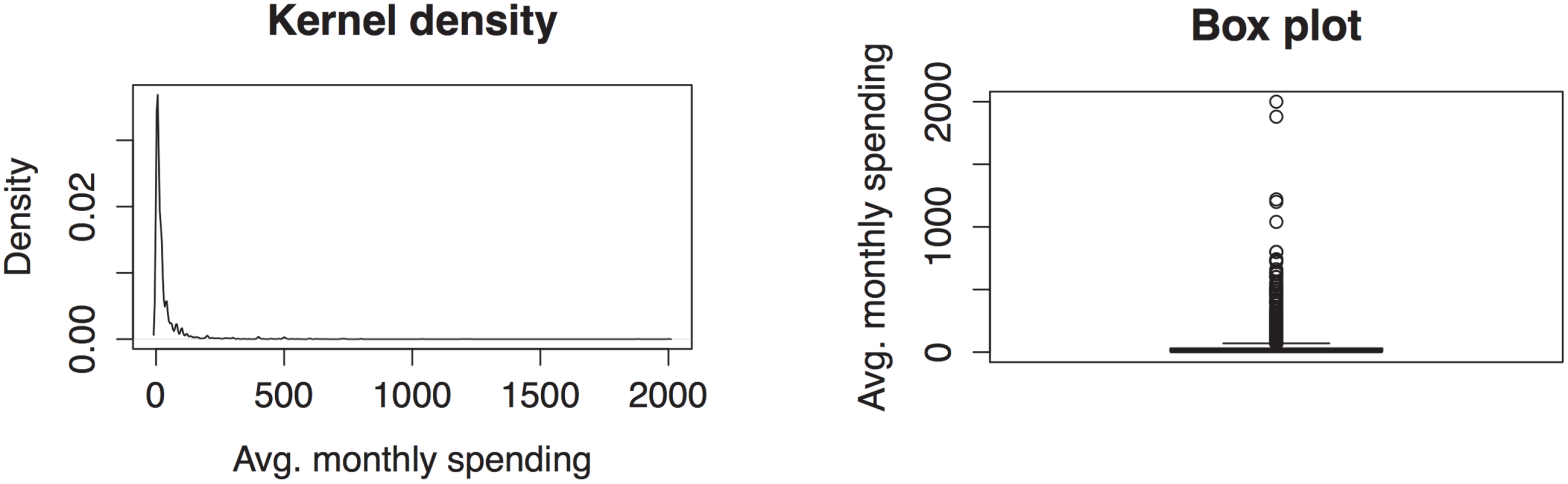
Kernel density plot and boxplot of nonzero values of reported lottery spending in an average month, obtained by combining the results of the Indiana, Gallup, and Texas surveys.

However, the previous work did not consider the peculiar shape of the distribution, which can be seen in Fig. 1. In both panels of the figure, the number being sampled is the answer to the question “How much do you spend on lottery tickets in a typical month?” and the data comes from the surveys [16–18]. The distribution has a lot of zeros—people who don’t play—and is positively skewed. Most people spend little to nothing on the lottery, but there are a few players who spend very large amounts, causing the tail seen in Fig. 1. The distribution look similar to classic examples of heavy-tailed distributions such as a Pareto. Other examples of empirical distributions with similar shapes are the populations of cities, the magnitude of earthquakes, and the Addiction Severity Index composite score as defined in [19].

## Methods

### Data sources

Among the many surveys that have asked respondents about their lottery participation, we focused on surveys that had two characteristics: First, they asked the question we wanted: “How much did you spend on lottery tickets in the last month?” or similar. For this reason, we excluded several otherwise-interesting surveys. Second, they were relatively recent. The 1989 book [2] and its 1999 sequel paper [7] are landmark references, so there seemed little point in mining 30-year-old surveys from the 1980s that preceded both works.

Consequently, we chose to analyze surveys from Texas (2007–2009 [16]) and Indiana (1990–1998 [18]) as well as a national survey performed by Gallup (January 2011 [17]). These surveys included 1617, 1359, and 365 gamblers, respectively. (Since we are only concerned with lottery spending, we use “gamblers” as a shorthand for “people who spend money on lottery tickets in a typical month”.)

### Parametric methods: problems with the data

**Fig 2:**
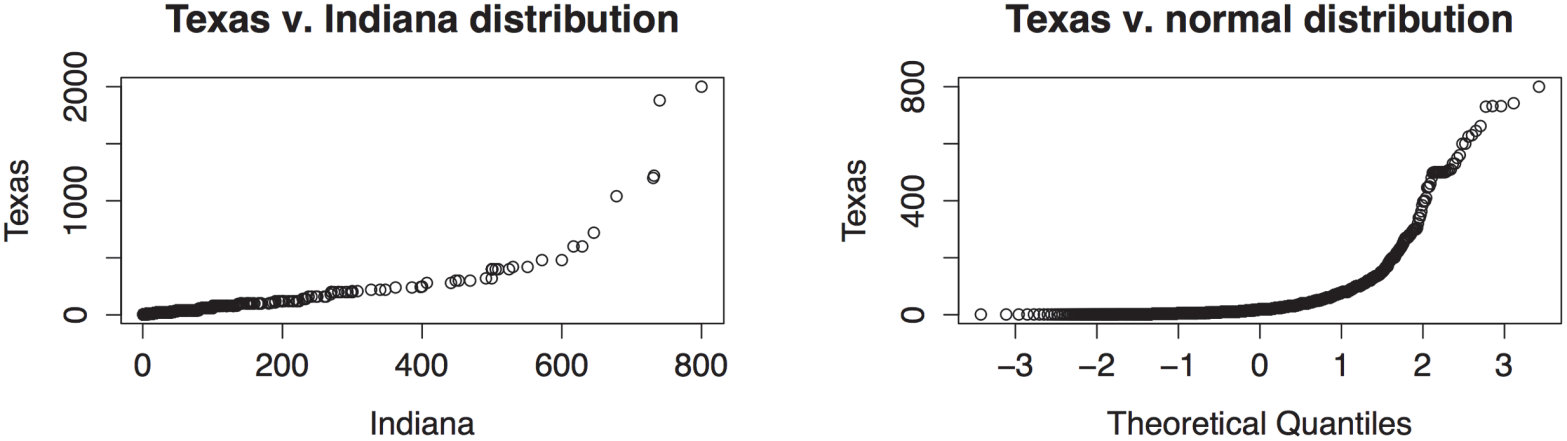
Q-Q Plots showing (left) that the data is different from survey to survey and (right) that the data is also non-normal. In each panel, if the two samples were drawn from the same distribution, the plotted points would lie approximately on the diagonal line joining the lower-left and upper-right corners.

Inspired by the paper [20], we had initially hoped to use the survey data to select a family of distributions that best fit the data, or at least the nonzero values in the data. However, an immediate problem presented itself in that the numbers from the various surveys do not appear to be from the same distributions, as is strongly indicated by the Q-Q plot in the left panel of Fig. 2.

Also, for the question we are interested in, the tail samples in this survey are obviously suspect: one imagines that the gamblers who spend the most on the lottery are also very likely to report a lower-than-actual amount of spending, and indeed such mis-reporting has been found to be typical, see [8] and [21].

Consequently, there is not much reason to believe that a probability distribution fit to the data would have much relation to the actual distribution of lottery spending. And it even seems unreasonable to use methods such as those described in Chapter 4 of [22] to give a parametric model for the tail, because these are the data points that are least certain. Therefore, we seek methods that are not highly sensitive to these difficulties with the data.

### Non-parametric methods

In view of the preceding discussion, we opted to employ non-parametric methods, as advocated in [23, 24], or see [25]. Specifically, we used *permutation tests* to compare the various distributions, as described in [26–28].

#### A toy example

To illustrate the notion of a permutation test, consider the toy example of a survey with two questions, “How much do you spend on lottery tickets in an average month?” and a question whose answer is a categorical variable with two possible values (such as gender), and suppose the 5 responses are:

$$\begin{array}{ccccccc} \text{spending}\;(\$) & | & 53 & 54 & 6 & 39 & 88\\ \text{categorical} & | & A & A & B & B & B \end{array}$$

We will consider the null hypothesis that the values of the real variable (spending) and the categorical variable are independent. Define a test statistic:

(average spending by A’s)−(average spending by B’s).

Which is approximately 9.2 for the actual survey data. It’s not zero, contrary to the null hypothesis, but is that significant? What is the p-value? Under the null, the value of the test statistic should not change if we randomly re-assign the categorical responses among the respondents. We randomly permute the A/B values among the 5 responses and for each permutation we re-calculate the test statistic. As an illustration, one of the permutations gives an alternative data set

$$\begin{array}{ccccccc} \text{spending}\;(\$) & | & 53 & 54 & 6 & 39 & 88\\ \text{categorical} & | & B & B & A & A & B \end{array}$$

on which the test statistic equals −42.5. Doing this for each of the 52=10 possible permutations, we find the following values of the test statistic, sorted into increasing order:

−42.5,−30.8,−30.0,−3.3,−2.5,−1.7,9.2,25.8,37.5,38.3. (1)

Suppose the alternative hypothesis is that A’s spend more than B’s, i.e., the test statistic is positive. (In our non-toy examples below, this will be provided by claims in the literature.) Then the p-value is the proportion of the numbers in (1) that are at least as large as the actual value, 9.2, of the test statistic. That is, the p-value is 4/10 = 0.4.

#### General procedure

Let us re-state this now in more abstract terms. Our data is in the form of survey responses, and we are interested in the case where each respondent gives a real number and a categorical variable with *c* possible values. (In the toy example, *c* = 2 and the real number is monthly lottery spending.) We consider the null hypothesis that the distribution of the real numbers is independent of the value of the categorical variable. We calculate, for the actual survey data, some test statistic, which will be a single number *t*_0_. In all our examples, this test statistic will be a real number and the test statistic will be designed so that under the null hypothesis it should be zero. In the *1-sided exact* permutation test, one considers the *N* ways of relabeling the categorical variables and for each of these re-computes the test statistic *t*_*j*_. The probability, then, that the null hypothesis holds and yet the test statistic *t*_0_ is so large is simply #{*j*∣*t*_*j*_ ≥ *t*_0_}/*N*, the number of relabelings for which the test statistic has a value at least as big as *t*_0_.

The precise number of relabelings, *N*, is given by the multinomial coefficient nn1,n2,…,nc=n!/(n1!n2!⋯nc!) where *n*_*i*_ is the number of responses in the *i*-th category. In practice this is quite large. The number *n* of responses in our entire pool is 3341 and the number *n*_1_ may be, say, 300, in which case already nn1 has more than 400 digits. Therefore it is not practical to perform the exact test. Instead, we perform a *Monte Carlo* permutation test by randomly sampling a large number *S* (say, 10^4^) of the *N* possible relabelings, compute the test statistic for each of these, and then report the p-value to be the proportion of these *S* values of the test statistic that are at least as large as *t*_0_. A 95% confidence interval can be obtained by reporting the 95th percentile of the test statistic among the *S* values.

## Results

### Comparison of demographic groups

As an illustration of our methods, we investigate the claim from Clotfelter-Cook:

In sum,members of certain groups are more likely to play lotteries and to play them heavily: males,Hispanics,blacks,the middle-aged,Catholics,laborers,and those with less than a college degree. (2)

This conclusion, perhaps, is not terribly surprising as these are approximately the same groups that are at elevated risk for pathological gambling, see [29–32]. Clotfelter and Cook looked at three measures of participation: participation rate (what fraction of survey respondents in a certain demographic group played the lottery), spending (comparing amount spent by gamblers in one demographic group to gamblers outside that group), and membership in the top 20% (comparing fraction of the top 20% of spenders coming from a particular demographic group, versus the fraction for the population as a whole). Because they focused on participation rates in their text discussion, we will focus on the other two measures; they claimed that the results were essentially the same for all three measures.

This is mostly well-trod ground, and we present results on these questions as an illustration of our different techniques in a context that will be familiar to some readers. Consequently, we mostly talk about those demographic groups that have been highlighted as heavier gamblers in previous studies: males, African Americans, Hispanics, and high school dropouts. We add in one new, interesting demographic axis: whether the survey respondent was reached on a cell phone or a land line. This information was included for respondents in both the Texas and Gallup surveys, providing spending numbers for 1982 gamblers.

#### Spending levels by gamblers from different groups

We use the previously-described 1-sided Monte Carlo 2-sample permutation test to test whether gamblers from a particular demographic group tend to have higher spending than gamblers who are not part of the group. We sample a random variable *X* from the demographic group that previous studies have shown to have higher spending (e.g., high school dropouts) and *Y* from the complement of this subgroup (e.g., gamblers who completed high school), and use for our test statistic the median of *X*−*Y* as *X* and *Y* range over both samples as described in §2.5 of [33] or Chapter 5 of [27]. Note that this test statistic, median(*X*−*Y*), is more interesting than and typically different from median(*X*)−median(*Y*), although in the case where the two populations have distributions that agree up to a location parameter, one can think of both as comparing the locations.

We use a 1-sided permutation test, because previous studies have claimed that this test statistic should be positive; the results are summarized in Table 1. While this reasoning does not apply to the cell phone vs. landline comparison, the unjustifiably-strong 1-sided test already gives a “statistically insignificant” p-value of 0.66, so a less powerful 2-sided test would not give any significant results.

**Table 1:** Spending levels by various demographic groups. The 95% confidence interval is the smallest median of difference one could find to produce a p-value of 0.05.

Demographic group	median of difference	est. p-value	95% confidence interval
Male	1	0.83	
African American	6	< 0.01	> 2
Hispanic	3	< 0.01	> 2
HS dropout	5	< 0.01	> 1
cell phone	2	0.66	

#### Membership in the top 20% for different groups

In the previous paragraph, we compared levels of spending by gamblers belonging to different demographic groups. Another way to compare the relative spending of demographic groups is to ask: is a group over-represented amongst the top 20% of lottery players? This has previously been investigated in [7], where it was found that males, African Americans, high school dropouts, and those with household income below $10,000 were overrepresented. Our results for similar categories are presented in Table 2.

**Table 2:** Representation of demographic groups in the top 20% of lottery players. P-values and confidence intervals are calculated using a binomial distribution based on the percentage of adults in the sample.

Demographic group	Percentage of adults in sample	Percentage of heaviest players	p-value	95% confidence interval
Male	50%	55%	0.005	≥ 53%
African American	11%	18%	< 0.001	≥ 13%
Hispanic	14%	20%	< 0.001	≥ 17%
HS dropout	5%	9%	< 0.001	≥ 7%
cell phone	15%	19%	0.011	≥ 18%

#### Why we ignored income

We ignored income in the demographic comparisons above for two reasons: first, there is the scientific reason that—contrary to popular perception that the poorer someone is, the more they tend to spend on the lottery—“the preponderance of the evidence suggests that there is little systematic relationship between income and the amount spent on lottery play” [2], p. 99. (See [34] for an illustration.) Second, there is the practical consideration that income is reported as a range, and the possible ranges differ from survey to survey.

### Controlling for confounding factors: Hispanic gamblers

We noted in Tables 1 and 2 that some African-American and Hispanic gamblers spend more on the lottery than gamblers outside those groups. But why should race or ethnicity be correlated with lottery spending?

Contrast this with low-education gamblers. We also found that high school dropouts spend more on the lottery than high school graduates, re-confirming part of a general relationship that has been widely observed, that more education means less spending on the lottery. This result, however, has a natural causal explanation, in that buying lottery tickets is not just a poor investment decision but also has a worse rate of return than other gambles such as roulette; we expect that a more educated gambler would therefore curtail their lottery purchases.

To look for explanations as to why being Hispanic (to take a particular example) might be correlated with higher lottery spending, we examined the Texas data, which had a large proportion of Hispanic gamblers. We found that the Hispanic gamblers in that sample tended to be less educated than the non-Hispanic gamblers. Might this correlation be what we are detecting when we note that “Hispanic gamblers spend more”?

To answer this question, we compared the spending of Hispanic and non-Hispanic gamblers while controlling for education. Using the non-parametric methods of this paper, we can do so by subdividing the population into blocks based on education—high school dropouts, those with only a high school diploma, those with some college, and those with a college degree—and comparing Hispanic to non-Hispanic gamblers within each block. For each block, we can compute the median of the difference *X*−*Y* as described in the previous section, where *X* is drawn from the spending of Hispanic gamblers in that block and *Y* is drawn from the non-Hispanic gamblers. We find this median is 0 for the high school dropouts, 2 for those with only a high school diploma, 2 for those with some college, and 0 for those with a college degree. The sum of these medians is 0+2+2+0 = 4, which is a measure of how much more Hispanic gamblers tend to spend than non-Hispanic gamblers, and it is not zero.

To assess whether the 4 was significant, we performed a permutation test where we only permuted the Hispanic/non-Hispanic labels within each block. For each permutation, we computed for each block the median of the difference *X*−*Y*, where *X* is drawn from the spending of Hispanic gamblers in that block and *Y* is drawn from the non-Hispanic gamblers (with respect to the permuted labels), and recorded the sum of these medians as the value of the test statistic. The null hypothesis is that being Hispanic (viewed as a convenient label for undetermined cultural factors) has no effect on lottery spending by itself, hence that the test statistic should be zero. We found that the true value, 4, of the test statistic has an approximate p-value of 0.3, so we have no reason to reject the null hypothesis at the 95% confidence level. That is, once we have controlled for education, we find no evidence that Hispanic gamblers spend more than non-Hispanic gamblers.

Let’s summarize this discussion. It is natural to believe that more education leads to spending less on the lottery, such a correlation has been widely observed in the literature, and we have observed it in our data as well. Once we control for this relationship, we find no evidence for increased spending by Hispanic gamblers. Occam’s Razor, then, says that *the increased spending by Hispanic gamblers appears to be due to their lower education as opposed to other, unspecified cultural factors*, up to an effect size that is too small to be detected with our techniques and data.

### Claims about heavy spenders

#### Percentage of spending due to heavy players

The book [2] by Clotfelter and Cook is a central reference in the subject of US lotteries. They write on p. 92:

Among those who do play,the top10percent of players in terms of frequency account for50percent of the total amount wagered,while the top20percent wager about65percent of the total. (3)

This is a specific example of what is sometimes called the “law of the heavy half” or Pareto’s law. Similar patterns can be found in the consumption of other goods; for example, a recent study, [35], found that the top 20% of marijuana consumers in Colorado accounted for about 65% of demand.

For the super-population obtained by combining our three main surveys, we found that the top 10% of players accounted for 58% of the total spending, and the top 20% of players accounted for 73%. These numbers are somewhat different from those claimed in (3).

#### Constancy of this percentage

Clotfelter and Cook follow (3) with a second claim:

Interestingly,the degree of concentration among players(as indicated by these percentages)does not depend on the time interval under consideration.Measures of concentration are virtually identical for three surveys that asked respondents to report lottery expenditures for some period preceding the interview: a one-week period(Maryland,1984),a two-month period(California,1985),and a twelve-month period(all lottery states combined,1974). (4)

We calculated these measures of concentration for our three surveys, and the results are displayed in Table 3. We can test the claim that these measures are constant across surveys by combining them into one super-population and performing a 3-sample permutation test where the test statistic is standard deviation of the measure of concentration, but with only 3 surveys it will take a large deviation from constancy to be detected in this way. Consequently, we include in this test all the surveys we found that asked for spending in a typical month, which we abbreviate as CBS (1989 [36], 607 gamblers), Kentucky (1989 [37], 332 gamblers), and Minnesota (1993, [38], 176 gamblers). (Concerning the claim (3), using all 6 surveys gives a super-population where the spending by the top 10% and 20% is nearly the same as was found using only the 3 most recent surveys—59% and 74% respectively.) We find that the standard deviations observed among the spending by the top 10% and by the top 20% differ from 0 with a p-value that is essentially 0, which refutes claim (4) that the percentage of spending due to the top 10 and 20 percent of players is essentially constant across surveys.

**Table 3:** Columns 2 and 3 report the % of total lottery spending due to the top 10% and 20% of spenders respectively. Column 4 reports the skewness and column 5 reports the skewness-adjusted kurtosis defined in [43] for the spending reported in each survey. The bottom three rows give the results of a 6-sample permutation test with test statistic the standard deviation of the numbers in the higher rows.

Survey	top 10%	top 20%	skewness	adj. kurtosis
Texas	55%	72%	4.2	3.3
Gallup	62%	75%	10.2	5.2
Indiana	56%	70%	10.5	7.0
CBS	45%	64%	3.4	3.1
Kentucky	38%	55%	4.5	4.3
Minnesota	63%	76%	6.3	4.2
Standard deviation	9.8%	7.8%	3.1	1.4
Estimated p-value	≈ 0	≈ 0	0.03	0.58
95% confidence interval	≥ 5.8%	≥ 4.2%	≥ 2.93	

### Higher moments

Another way to measure how far the distribution of spending deviates from normal is to consult the higher moments, namely the skewness and excess kurtosis, which are defined as follows. For *X* a random variable with mean *μ*, one defines the centralized *j*-th moment $\mu_{j}:=E[{\left(X-\mu \right)}^{j}]$, so the standard deviation is $\sigma :=\sqrt{\mu_{2}}$. The skewness *α*_3_ and kurtosis *α*_4_ of *X* are the normalized *j*-th moments defined by *α*_*j*_: = *μ*_*j*_/*σ* ^*j*^. A normally distributed random variable has skewness 0 and kurtosis 3, regardless of its mean and standard deviation.

#### Skewness

We provide in Table 3 the skewness for the distribution of spending in various surveys; clearly, all are positively skewed. Applying a permutation test shows the standard deviation of these skewness numbers will arise by chance with estimated probability 3%, so there is no reason to think that skewness is consistent across surveys. For demographic groups, we asked if skewness captures a difference in the nature of spending by one demographic group relative to those who are not members of the group; applying two-sample permutation tests with the demographic groups considered in Tables 1 and 2 did not detect any significant difference.

#### Kurtosis

As for kurtosis, it is commonly believed that a symmetric distribution with high kurtosis—i.e., that is “leptokurtotic”—has heavier tails. (The paper [39] says that kurtosis “can be vaguely defined as the location- and scale-free movement of probability mass from the shoulders of a distribution into its center and tails.”) The distribution of spending in all of the surveys in Table 3 is highly leptokurtotic (some have kurtosis of over 100) and the kurtosis varies from survey to survey. Neither of these are surprising in view of the skewness discussion in the previous paragraph, as skewness and kurtosis are related, for example by the inequality $\alpha_{4}\geq \alpha_{3}^{2}+1$ noticed already in [40]. Consequently, it would make sense to use instead a “skewness-adjusted” version of *α*_4_. We investigated various options such as those introduced in [41–43] and surveyed in [39], but we did not find any clearly significant results. For the curious reader, we report the modified kurtosis from [43] in Table 3; this measure produced the least-varying numbers of the most civilized magnitudes.

## Discussion

We applied a non-parametric method (permutation test) to investigate which demographic groups spend more on the lottery and whether the proportion of spending by big spenders is constant (as was claimed in the literature). We applied this method to more recent data than previous studies, and we found confirmation of similar demographic claims. We found strong evidence that gamblers that were African-American, were Hispanic, or did not finish high school spent more on lottery tickets than gamblers that were not in these groups, and that these three categories of gamblers were overrepresented among the top 20% of lottery players by spending. This agrees with what has been found in previous studies.

Although it may appear that we have found weak evidence that male gamblers and cell phone respondents appeared more frequently among the top 20% of players, because we are doing at least 10 statistical tests, a p-value of 0.005 is not necessarily significant.

While we saw that the top 10% of spenders typically account for more than half of all sales, we refuted the claim that the amounts spent by the top 10% and by the top 20% of spenders are constant across US lottery surveys.

We also found no evidence that higher spending by Hispanic gamblers is a reflection of unspecified cultural factors having to do with race; instead, their higher spending appears to be accounted for by their lower levels of education relative to non-Hispanic gamblers. This observation seems to be new.
